# Melatonin Alleviates Hypoxia-Induced Apoptosis of Granulosa Cells by Reducing ROS and Activating MTNR1B–PKA–Caspase8/9 Pathway

**DOI:** 10.3390/antiox10020184

**Published:** 2021-01-28

**Authors:** Jing-Li Tao, Xuan Zhang, Jia-Qi Zhou, Cheng-Yu Li, Ming-Hui Yang, Zhao-Jun Liu, Liang-Liang Zhang, Shou-Long Deng, Lu Zhang, Ming Shen, Guo-Shi Liu, Hong-Lin Liu

**Affiliations:** 1 College of Animal Science and Technology, Nanjing Agricultural University, Nanjing 210095, China; taojingli@njau.edu.cn (J.-L.T.); 2018105015@njau.edu.cn (X.Z.); 2018105014@njau.edu.cn (J.-Q.Z.); 2018205006@njau.edu.cn (C.-Y.L.); 2018105080@njau.edu.cn (Z.-J.L.); 2018105016@njau.edu.cn (L.-L.Z.); shenm2015@njau.edu.cn (M.S.); 2 National Engineering Laboratory for Animal Breeding, Key Laboratory of Animal Genetics and Breeding of the Ministry of Agriculture, College of Animal Science and Technology, China Agricultural University, Beijing 100193, China; Yangmh17@cau.edu.cn (M.-H.Y.); luzhang2018@cau.edu.cn (L.Z.); gshliu@cau.edu.cn (G.-S.L.); 3 CAS Key Laboratory of Genome Sciences and Information, Beijing Institute of Genomics, Chinese Academy of Sciences, Beijing 100101, China; dengsl@big.ac.cn

**Keywords:** melatonin, hypoxia, granulosa cells, apoptosis, reactive oxygen species

## Abstract

In mammalian ovaries, the avascular environment within follicular cavity is supposed to cause hypoxic status in granulosa cells (GCs), leading to apoptotic cell death accompanied by cumulative reactive oxygen species (ROS) production. Melatonin (N-acetyl-5-methoxytryptamine, MT), a broad-spectrum antioxidant that exists in porcine follicle fluid, was suggested to maintain GCs survival under stress conditions. In this study, using the established hypoxic model (1% O_2_) of cultured porcine GCs, we explored the effect of MT on GCs apoptosis. The results showed that MT restored cell viability and reduced the apoptosis of GCs during hypoxia exposure. In addition, GCs treated with MT exhibited decreased ROS levels and increased expression of antioxidant enzymes including heme oxygenase-1 (HO-1), glutathione S-transferase (GST), superoxide dismutase 1 (SOD1), and catalase (CAT) upon hypoxia incubation. Moreover, the hypoxia-induced expression of cleaved caspase 3, 8, and 9 was significantly inhibited after MT treatment. In contrast, blocking melatonin receptor 2 (MTNR1B) with a competitive antagonist 4-phenyl-2-propionamidotetralin (4P-PDOT) diminished the inhibitory effects of MT on caspase 3 activation. By detecting levels of protein kinase (PKA), a downstream kinase of MTNR1B, we further confirmed the involvement of MT–MTNR1B signaling in mediating GCs protection during hypoxia stress. Together, the present data provide mechanistic evidence suggesting the role of MT in defending GCs from hypoxia-induced apoptosis.

## 1. Introduction

In mammalian, more than 99% of the ovarian follicles undergo atresia during development [1,2]. Actually, apoptosis of ovarian granulosa cells (GCs) occurs much earlier than the morphological changes of follicular atresia, which could be observed only when GCs apoptosis reaches a certain level. Therefore, GCs apoptosis is considered to be the initiating factor leading to follicular atresia [3,4]. In atretic follicles, the GCs showed typical features of apoptotic death, such as nuclear pyknosis and DNA fragmentation [5]. The identification of internucleosomal DNA fragmentation during atresia in porcine ovaries suggests that apoptosis is a basic mechanism associated with ovarian follicular atresia in mammalian as well as avian species [5]. Hereafter, the phenomenon of GCs apoptosis has also been detected in atretic follicles of mice, pigs, cattle, sheep, and other animals [6,7,8,9].

Follicular blood vessels provide essential nutrients, hormones, and oxygen (O_2_) required for the development of ovarian follicles. However, the follicular capillaries are restrictedly distributed in the theca layers outside the membrane propria, creating an avascular environment within the follicular wall, thus limiting the accessibility of O_2_ to GCs. During follicular development, the growing distance from GCs to blood vessels further exacerbate the hypoxia conditions [10]. It was reported that the decrease in oxygen partial pressure (*p*O_2_) could increase superoxide anion (O_2_^−^) production by activating NAD(P)H oxidase [11]. Recent studies confirmed that ROS was produced in a large amount during follicular development. Gupta et al. found that the average level of H_2_O_2_ exceeds 1000 ng/mL in the follicular fluid [12]. In humans, the average concentration of ROS was more than 95cps (counted photons per second) per 400 µL follicular fluid in healthy follicles [13]. Giuseppina et al. reported that more than 7.5 µM of H_2_O_2_ was detected in small, medium, and large follicles in porcine ovary [10].

The excessive ROS was known to cause oxidative stress. A bundle of evidence suggests that oxidative stress exerts a negative influence on follicular development. Matzuk et al. reported that the number of pre-ovulatory follicles and corpus luteum on the ovaries of adult female mice was reduced after knocking out superoxide dismutase (SOD1) [14]. The inhibition of glutathione (GSH) synthesis in mice ovaries leads to oxidative damage of GCs, thereby accelerating ovarian aging and the resultant decline in reproductive ability [15]. Compared with healthy follicles, the level of apoptosis and protein oxidation in atretic follicles increased, while the content of GSH decreased [16]. Indeed, using an ovarian-specific oxidative stress mouse model, we previously found that oxidative stress significantly promoted ovarian GCs apoptosis and follicular atresia [17,18]. On the contrary, inhibiting the ROS production in follicles significantly restrained GCs apoptosis and follicular atresia caused by oxidative stress [19,20]. These findings further support that oxidative stress is an important cause of follicular atresia and GCs apoptosis.

Melatonin (N-acetyl-5-methoxytryptamine, MT) is a pleiotropic molecule that regulates diverse cellular and physiological processes. Owing to the potent antioxidation activity, MT is widely studied in the redox biology. It is worth noting that MT concentration in human follicular fluids was three-fold higher than in serum [21,22]. MT was also detected in porcine follicular fluid [23]. MT concentration was significantly decreased as follicular atresia progressed, which is accompanied by increased GCs apoptosis. He et al. found that MT could prevent GCs apoptosis during follicular atresia via its membrane receptors and its free-radical-scavenging activity [24]. Actually, MT functions by directly scavenging toxic oxygen derivatives [25], reducing ROS generation [26], or indirectly by stimulating the activity of anti-oxidative enzymes [27], including glutathione peroxidase (GSH) and superoxide dismutase (SOD) [28]. MT has been shown to inhibit apoptosis by eliminating oxidative stress, thereby blocking the downstream mechanism that triggers apoptosis [29]. However, it remains elusive whether MT exerts any influence on GCs survival under hypoxia conditions. In this study, we demonstrated the anti-apoptotic role of MT in hypoxic GCs via eliminating ROS and activating the MTNR1B–PKA–caspase8/9 pathway.

## 2. Materials and Methods

### 2.1. Ethics Statement

All experiments and treatments were approved and supervised by the Animal Research Institute Committee of Nanjing Agricultural University (Permit Number: IACUC2020132), China.

### 2.2. Reagents and Antibodies

DMEM/F12, fetal bovine serum (FBS), and phosphate buffered saline (PBS) were purchased from Gibco (Grand Island, NY, USA). Melatonin (M5250), Luzindole (L2407), 4P-PDOT (SML1189), and SR1001 (SML0322) were purchased from Sigma-Aldrich (St. Louis, MO, USA). Z-IETD-FMK (S7314), Z-LEHD-FMK (S7313), and apoptosis activator (S2927) were purchased from Selleck Chemicals (Houston, TX, USA). Antibodies against caspase-3 (19677-1-AP) were purchased from Proteintech Group (Chicago, IL, USA). Antibodies against caspase-8 (9508), caspase-9 (4790), and TUBA1A (2125) were purchased from Cell Signaling Technology (Beverly, MA, USA). An Annexin V-FITC Apoptosis Detection Kit (A211) was purchased from Vazyme (Nanjing, China). A Fluorometric Intracellular ROS Kit (MAK145) was purchased from Sigma-Aldrich (St. Louis, MO, USA). A protein kinase (PKA) enzyme linked immunosorbent assay (ELISA) Kit was purchased from Lengton (Shanghai, China).

### 2.3. Cell Culture and Treatments

Porcine ovaries were collected from the local abattoir (Nanjing, China) and transported immediately to the laboratory within 2 h in the solution of PBS (32 °C) supplemented with 500 IU/mL penicillin–streptomycin (Sigma-Aldrich, P7794 and S1277). Ovaries were soaked for 30 s using 75% ethyl alcohol and washed several times in PBS. Granulosa cells (GCs) were aspirated from the follicles (3–5 mm in diameter) using a 10-mL syringe fixed with an 18-gauge needle. GCs were washed three times in PBS and then were cultured in DMEM/F12 medium with 10% FBS plus 1% penicillin–streptomycin at 37 °C in a humidified atmosphere of 5% CO_2_ for 36 h. For drug administration, porcine GCs were treated with different MT concentrations (0, 10^−9^, 10^−7^, 10^−5^, 10^−3^ M) or inhibitors. For hypoxia environment, GCs were subjected to hypoxic conditions (1% O_2_, 5% CO_2_, balanced N_2_).

### 2.4. Western Blotting

Total protein was extracted from treated GCs using ice-cold RIPA (Radio Immunoprecipitation Assay) Buffer (Beyotime, Shanghai, China) supplemented with 1 mM phenylmethylsulfonyl fluoride (PMSF; Beyotime, Shanghai, China). The protein concentrations were measured using a BCA (bicinchoninic acid) Protein Assay Kit (Beyotime, Shanghai, China). Protein denaturation was performed via boiling cell lysates for 10 min in SDS loading buffer (biosharp, Shanghai, China). Equal amounts of proteins were resolved using 12% SDS-PAGE gel and transferred to PVDF (polyvinylidene difluoride) membranes (Millipore, Bedford, MA, USA). After they were blocked with 5% non-fatty milk at 37 °C for 60 min, the membranes were incubated with primary antibodies against caspase-3 (1:1000, Proteintech, 19677-1-AP), caspase-8 (1:1000, Cell Signaling Technology, 4790), caspase-9 (1:1000, Cell Signaling Technology, 9508), and TUBA1A (1:1000, Cell Signaling Technology, 2125) at 4 °C overnight. Then, the membranes were washed three times with TBST (Tris buffered saline with Tween 20) buffer and incubated with horseradish peroxidase (HRP)-conjugated secondary antibodies (Goat Anti-Rabbit IgG (immunoglobulin G) H&L (HRP), 1:2000, Abcam, ab6721; Goat Anti-Mouse IgG H&L (HRP), 1:2000, Abcam, ab6789). Then, protein bands were detected by chemiluminescence. Band analyzed by Image J software (version 1.45; National Institutes of Health, Bethesda, MD, USA) and the values for target proteins were normalized to TUBA1A as the endogenous control.

### 2.5. Apoptosis by Flow Cytometry Analysis

The apoptosis rate of porcine GCs was measured using an Apoptosis Detection Kit (Vazyme Biotech) according to the manufacturer’s protocol. In total, 2 × 10^4^ cells were sorted by FACS (fluorescence activated Cell Sorting) using a cell counting machine (Becton Dickinson, Franklin, NJ, USA). The apoptosis rate was calculated using the following equation: (number of cells in the right upper quadrant + number of cells in the right lower quadrant)/(total number of cells).

### 2.6. Cell Viability Assay

The cell viability of GCs was measured using a Cell Counting Kit-8 (CCK-8; Dojindo Laboratories, CK04), in which the tetrazolium salt (WST-8) is reduced by dehydrogenase activities in viable cells to generate a yellow water-soluble formazan dye. Therefore, the intensity of color is directly proportional to the number of living cells in culture. The experimental procedures were carried out following the manufacturer’s directions. Briefly, GCs were seeded in 96-well plates and grown to 90% confluency for 3 days. After the indicated treatments, CCK-8 assay reagent (10 μL) was added to each well containing 100 μL medium and incubated in the dark for 2 h at 37 °C. The formation of formazan was assessed by determining the optical density (OD) at 450 nm under a microplate spectrophotometer (Thermo Fisher Scientific, Camarillo, CA, USA).

### 2.7. Detection of ROS by Immunofluorescence-Confocal Microscopy and Flow Cytometry Analysis

ROS (reactive oxygen species) levels were determined using the Fluorometric Intracellular ROS Kit (MAK145) according to the manufacturer’s instructions. The Fluorometric Intracellular ROS Assay Kit provides a sensitive, one-step fluorometric assay to detect intracellular ROS (especially superoxide and hydroxyl radicals) in live cells within 1 h of incubation. ROS react with a cell-permeable sensor, resulting in a fluorometric product (lex = 520/lem = 605 nm) proportional to the amount of ROS present. Ready-to-use DAPI (4′,6-Diamidino-2-phenylindole dihydrochloride, KGA215-50) were purchased from KeyGEN BioTECH (Jiangsu, China). The cells were imaged with a laser-scanning confocal microscope (Carl Zeiss, Zeiss LSM 710 META, Oberkochen, Germany). The results were calculated as fluorescence intensity in each GC by using the ImageJ 1.42q software (National Institutes of Health, Bethesda, MD, USA).

ROS levels were also detected by Flow Cytometry Analysis (FACS Calibur, American BD) according to the manufacturer’s instructions. Briefly, add 100 μL/well (96-well plate) of Master Reaction Mix into the cell plate. Incubate the cells in a 5% CO_2_, 37 °C incubator for one hour. Treat cells with 20 μL/well of test compounds in suitable buffer. Measure the fluorescence intensity using a BD Accuri C6 flow cytometer. The data were analyzed using the FlowJo v7.6 software (Stanford University, Stanford, CA, USA).

### 2.8. Real-Time Quantitative RT-PCR Analysis

Total RNA was isolated with TRIzol reagent (Invitrogen, USA) and was immediately reverse-transcribed using Prime Script™ RT reagent Kit with gDNA Eraser (TaKaRa Bio Inc., RR047). The abundance of *HO-1, TRX1, GST, SOD1, SOD2, CAT,* and *GPX4* mRNA molecules was measured by qRT-PCR. *GAPDH* and *β-actin* was used as housekeeping genes. The primers are shown in Table 1. Relative mRNA expression was calculated by the 2^−ΔΔCT^ method.

### 2.9. PKA Detection

We used the PKA ELISA Kit (Shanghai, China) to detect the PKA content according to the manufacturer’s instructions. Briefly, we added the sample to the pre-coated enzyme-labeled wells; then we added the biotin-labeled recognition antigen and incubated at 37 °C for 30 min. Then, we washed the same with PBST (phosphate buffered saline with 0.05% Tween 20) to remove unbound biotin antigen; then, we added avidin–HRP and incubated at 37 °C for 30 min. After washing, the absorption peak at 450 nm was assessed under a microplate spectrophotometer (Thermo Fisher Scientific, Camarillo, CA, USA).

PKA activity assay. PKA activity was examined using the PKA Kinase Assay Kits, Type I (Immunechem) according to the manufacturer’s protocol. In brief, porcine ovarian GCs were collected in 150 μL PBS and were lysed by repeatedly freezing and thawing. Then, we transferred the supernatant to an EP (Eppendorf) tube after centrifugation. The supernatant (30 μL/well) was added into the substrate plate containing kinase assay dilution buffer. Adenosine triphosphate (ATP) (10 μL/well) was added to initiate the kinase reaction at 30 °C for 90 min. After the reaction solution was removed, anti-p-substrate antibodies (40 μL/well) were incubated for 60 min at room temperature. Goat anti-rabbit IgG HRP was used as secondary antibody. TMB (3, 3′,5, 5′-Tetramethylbenzidine) solution was used to develop the color, indicating reaction activity. OD450 was detected to calculate the relative kinase activity. The enzyme activity was normalized to the protein concentration, which was measured by using the BCA method.

### 2.10. Statistics Analysis

All experiments were repeated at least three times. Data were expressed as mean ± S.E.M. Statistical analyses were done using the univariate analysis of variance (ANOVA) followed by the Student t-test with SPSS 21.0 statistical software. *p* < 0.05 was considered significant, and *p* < 0.01 was considered highly significant.

## 3. Results

### 3.1. Melatonin Reduced Apoptosis and Rescued Cell Viability of Porcine Granulosa Cells (GCs) in the Setting of Hypoxia

**Figure 1:**
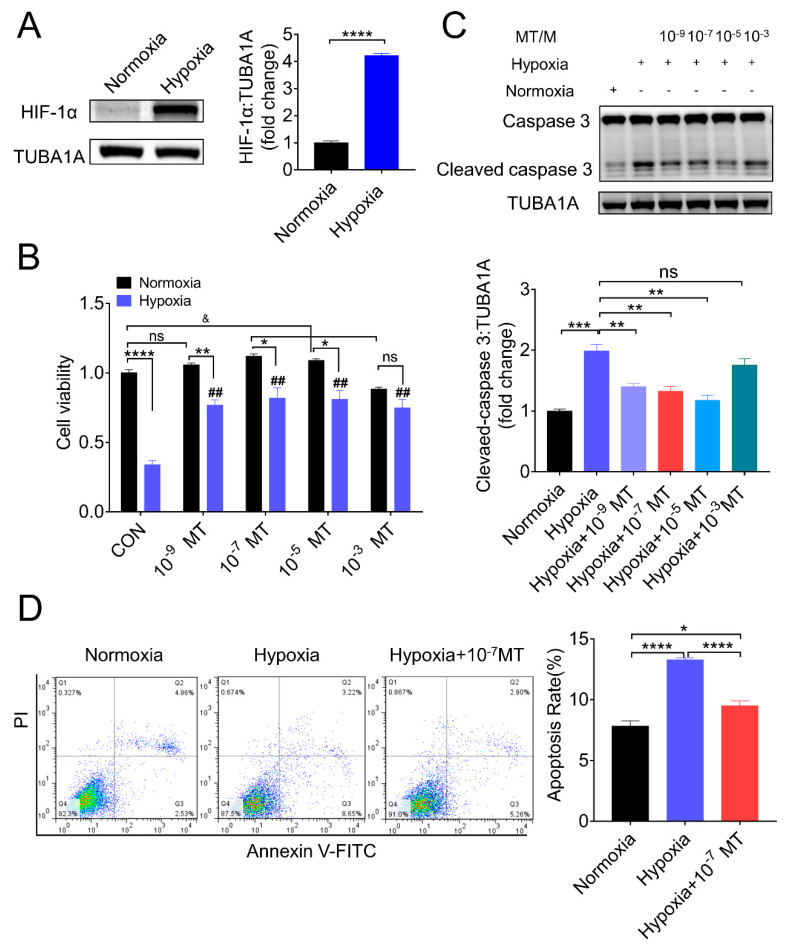
Effect of melatonin (MT) on porcine granulosa cells (GCs) viability and apoptosis during hypoxia. (**A**) The expression of hypoxia-inducible factor 1α (HIF-1α) determined by Western blotting in GCs cultured in normoxia and hypoxia. (**B**) The cell viability of porcine GCs grown in normoxia and hypoxia in the presence of different concentrations of MT (0, 10^−9^, 10^−7^, 10^−5^, 10^−3^ M). (**C**) The level of caspase−3 and cleaved caspase−3 determined by Western blotting in GCs grown in normoxia and hypoxia in the presence of different concentrations of melatonin (0, 10^−9^, 10^−7^, 10^−5^, 10^−3^ M). (**D**) Flow cytometry analysis of the apoptosis of porcine GCs. * *p* < 0.05 was considered significant; ** *p* < 0.01 was considered highly significant; *** *p* < 0.001 was considered very significant; **** *p* < 0.0001 was considered extremely significant; ^##^ *p* < 0.01 was considered highly significant; ^&^ *p* < 0.05 was considered significant; ^ns^ *p* > 0.05 was considered no significant.

Using an established in vitro hypoxic model [30,31], we verified the hypoxia state by detecting the protein level of hypoxia-inducible factor 1α (HIF-1α), which showed a marked upregulation in GCs cultured with 1% O_2_ (Figure 1A). Concomitantly, the cell viability was significantly decreased after hypoxia incubation, along with an increased level of cleaved caspase-3 (Figure 1B,C). To detect if melatonin could counteract the detrimental effect of hypoxia, porcine GCs were treated in normoxia and hypoxia (1% O_2_) supplemented with different MT concentrations (0, 10^−9^, 10^−7^, 10^−5^, 10^−3^ M). As shown in Figure 1B,C, MT concentration at 10^−9^, 10^−7^, and 10^−5^ M significantly restored GCs viability and reduced the level of cleaved caspase-3 induced by hypoxia. Through flow cytometry analysis, we further affirmed that MT at 10^−7^ M attenuated the apoptosis of porcine GCs triggered by hypoxia (Figure 1D).

### 3.2. Melatonin Lowered ROS Level Caused by Hypoxia in Porcine GCs

**Figure 2:**
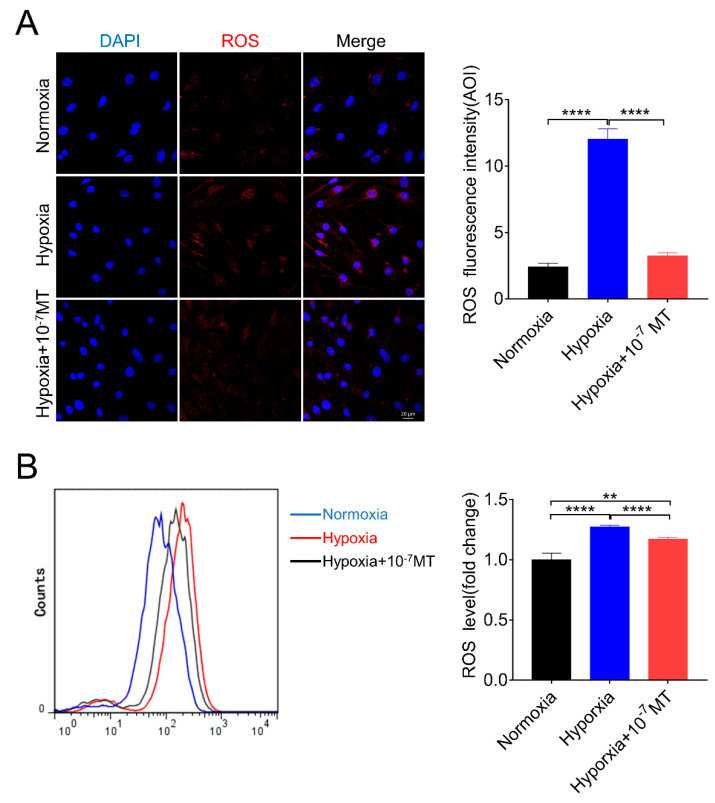
Effect of MT on reactive oxygen species (ROS) levels of porcine GCs in normoxia and hypoxia environment. (**A**) The immunofluorescence analysis of ROS levels in porcine GCs cultured in normoxia, hypoxia, and hypoxia plus 10^−7^ M MT. (**B**) The flow cytometry analysis of ROS levels of porcine GCs treated in normoxia, hypoxia, and hypoxia plus 10^−7^ M MT. ** *p* < 0.01 was considered highly significant; **** *p* < 0.0001 was considered extremely significant.

Since hypoxia environment has been implicated in triggering ROS production, we next investigated whether MT functioned by decreasing ROS content in hypoxic GCs. As shown in Figure 2A, we found that hypoxia caused an increase in ROS level, which was reduced after MT treatment. Using flow cytometry, we further confirmed the role of MT in suppressing ROS levels during hypoxia (Figure 2B).

### 3.3. Melatonin Suppressed Hypoxia-Induced Apoptosis of Porcine GCs by Eliminating ROS and Increasing the Expression of Antioxidant Enzymes

**Figure 3:**
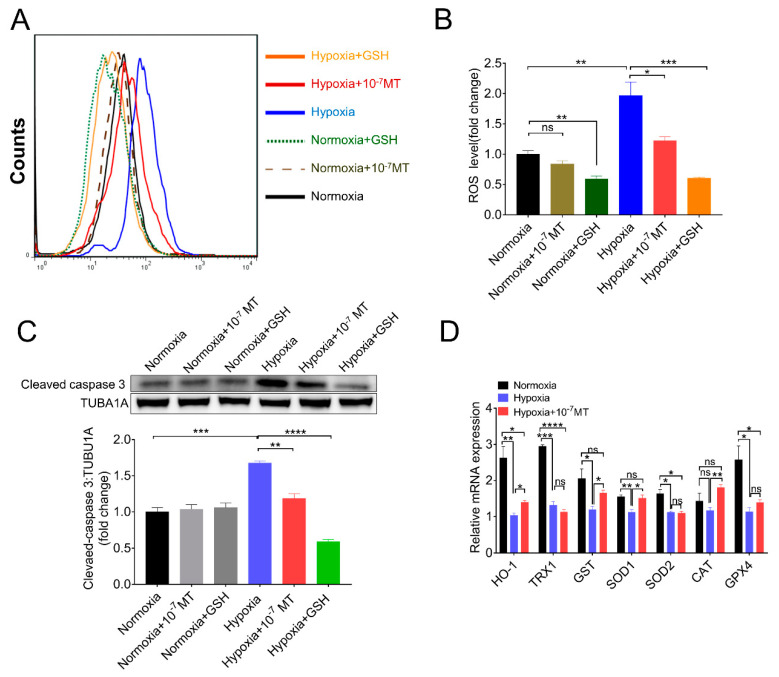
MT-mediated GCs protection during hypoxia is partly dependent on its antioxidant activity. (**A**) The flow cytometry analysis of ROS levels of porcine GCs. (**B**) Quantitative analysis of ROS levels. (**C**) The Western blot analysis of cleaved caspase−3 in porcine GCs treated with MT and glutathione (GSH). (**D**) The relative mRNA expression level of antioxidant enzymes of porcine GCs cultured in normoxia, hypoxia, and hypoxia plus 10^−7^ M MT. * *p* < 0.05 was considered significant; ** *p* < 0.01 was considered highly significant; *** *p* < 0.001 was considered very significant; **** *p* < 0.0001 was considered extremely significant; ^ns^ *p* > 0.05 was considered no significant.

MT could directly remove ROS or indirectly serve a function by increasing the expression of antioxidant enzymes and GSH [32,33]. GSH, the tripeptide, usually considered, has the most important functions for the removal of peroxides. Thus, we use GSH as the positive control of scavenging ROS. As shown in Figure 3A,B, both MT and GSH lowered ROS levels induced by hypoxia. MT treatment also revealed less inhibitory effect on caspase-3 activation compared with GSH (Figure 3C), suggesting that MT-suppressed GCs apoptosis might be partially achieved via directly removing ROS. By determining the mRNA levels of antioxidant enzymes, we found that MT rescued the expression of heme oxygenase-1 (HO-1), glutathione S-transferase (GST), superoxide dismutase 1 (SOD1), and catalase (CAT) under hypoxia condition (Figure 3D).

### 3.4. Melatonin Alleviated Hypoxia-Induced Apoptosis by Deactivating Caspase-8 and Caspase-9

**Figure 4:**
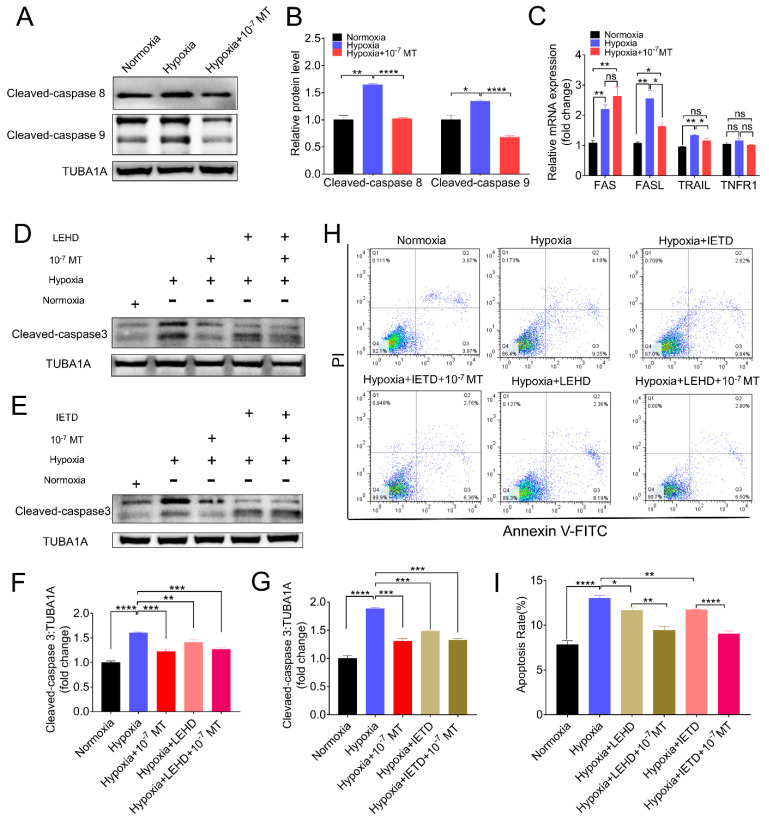
The suppression of caspase−8 and caspase−9 is required for MT-mediated GCs protection against hypoxia-induced apoptosis. (**A**) The level of cleaved caspase−8 and cleaved caspase−9 was determined by Western blotting in porcine GCs cultured in normoxia, hypoxia, and hypoxia plus 10^−7^ M MT. (**B**) Quantitative analysis of the level of cleaved caspase−8 and cleaved caspase−9. (**C**) The relative mRNA expression level of the receptor and ligand of extrinsic apoptotic pathways. (**D**,**E**) The level of cleaved caspase−3 was determined by Western blotting in GCs cultured in normoxia and hypoxia in the presence of MT, LEHD (Z-LEHD-FMK, caspase−9 inhibitor), or IETD (Z-IETD-FMK, caspase−8 inhibitor). (**F**,**G**) Quantitative analysis of cleaved caspase−3 level. (**H**) Flow cytometry analysis of the apoptosis rates in porcine GCs treated with IETD. (**I**) Statistics of the apoptosis of porcine GCs. * *p* < 0.05 was considered significant; ** *p* < 0.01 was considered highly significant; *** *p* < 0.001 was considered very significant; **** *p* < 0.0001 was considered extremely significant; ^ns^ *p* > 0.05 was considered no significant.

Apoptosis is a strictly regulated process activated by extrinsic or intrinsic pathways. Each pathway activates its own initiator caspase (8, 9), which in turn will activate the executioner caspase-3 [34]. As shown in Figure 4A,B, hypoxia induced the expression of both caspase-8 and caspase-9, which was reversed following MT administration. To further assess whether caspase-8 and caspase-9 affected the pro-survival actions of MT under hypoxia conditions, GCs were treated with caspase-8 and caspase-9 inhibitor Z-IETD-FMK (IETD, Z-Ile-Glu(O-ME)-Thr-Asp(O-Me) fluoromethyl ketone) and Z-LEHD-FMK TFA (LEHD, Z-Leu-Glu(O-Me)-His-Asp(O-Me) fluoromethyl ketone trifluoroacetate salt hydrate), respectively. As shown in Figure 4D–G, both LEHD and IETD decreased the level of cleaved caspase-3, and they displayed similar preventive effects with MT on hypoxia-induced apoptosis of porcine GCs. Using flow cytometry analysis, we obtained consistent results that MT, LEHD, and IETD decreased apoptosis rates in hypoxic GCs (Figure 4H,I). We also examined the receptor and ligand of extrinsic apoptotic pathways using qRT-PCR. The results showed that MT decreased the expression of FASL and TRAIL induced by hypoxia (Figure 4C).

### 3.5. Melatonin Inhibited Hypoxia-Induced Apoptosis via MTNR1B-PKA Pathway

**Figure 5:**
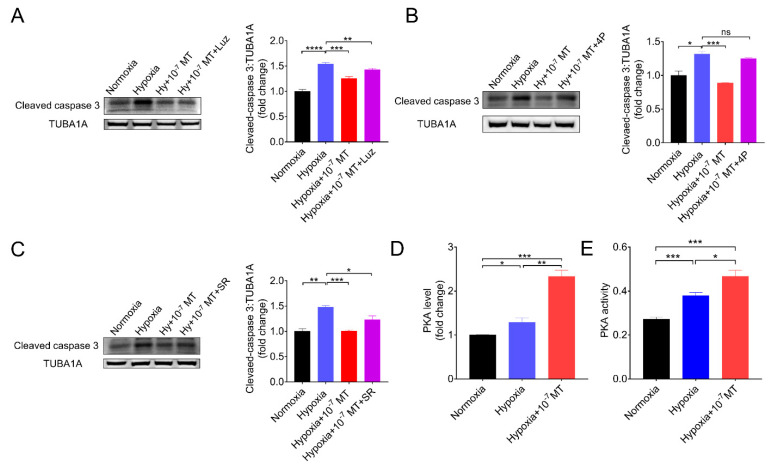
MT inhibited hypoxia-induced GCs apoptosis by binding to its receptor MTNR1B. (**A**–**C**) The level of cleaved caspase−3 was determined by Western blotting in GCs cultured in normoxia and hypoxia in the presence of 10^−7^ M MT, Luzindole (Luz) (**A**), 4P-PDOT (4P) (**B**), or SR1001 (SR) (**C**). (**D**,**E**) The determination of PKA concentration (**D**) and activity (**E**) in normoxia, hypoxia, and hypoxia plus 10^−7^ M MT. * *p* < 0.05 was considered significant; ** *p* < 0.01 was considered highly significant; *** *p* < 0.001 was considered very significant; **** *p* < 0.0001 was considered extremely significant; ^ns^ *p* > 0.05 was considered no significant.

The physiological effects of MT are mediated through binding with its membrane receptors (MTNR1A, MTNR1B), nuclear binding sites (ROR), or non-receptor pathways. In current research, using MT membrane receptor antagonists (Luzindole and 4P-PDOT) and a selective RORα and RORγ inhibitor (SR1001), we further assessed the protective effects of MT on GCs under hypoxia. As shown in Figure 5A–C, 4P-PDOT, a competitive antagonist of MTNR1B, completely negated the action of MT on inhibiting caspase-3 cleavage during hypoxia. Since PKA is a downstream effector of MTNR1B, we then detected PKA activation after MT treatment. The result showed that the concentration and activity of PKA in hypoxic GCs was further increased after MT treatment (Figure 5D,E).

## 4. Discussion

The avascular environment within ovarian follicles limits oxygen supply for GCs, and the hypoxia status of the GCs are gradually intensified as the follicle develops, during which a large amount of growing follicles undergo degeneration via atresia. In fact, our previous studies have demonstrated that under severe hypoxia stimulation, increased apoptosis occurred in ovarian GCs [30,31], indicating that hypoxia may be an important factor in initiating follicular atresia. Interestingly, some of the follicles retain the potential to develop toward ovulation. However, it remains to be uncovered whether certain intra-ovarian factors preserved the viability of GCs during hypoxia stress. In this study, we found that (1) MT inhibited hypoxia-induced apoptosis of GCs. (2) MT mitigated oxidative stress, increased the expression of antioxidant enzymes, and reduced apoptosis in hypoxic GCs. (3) MT acts through receptor MTNR1B to block the activation of caspase-8/9–caspase-3 axis, thereby alleviating the apoptosis of GCs. To our knowledge, these findings provide evidence suggesting a role of MT in maintaining GCs survival and follicular development under hypoxia.

MT is a ubiquitous hormone primarily produced by the pineal gland in the circulating system and locally synthesized in several cells and tissues [35,36]. In the ovary, granulosa cells, cumulus cells, and oocytes have been reported to synthesize melatonin [37,38,39], which might function in concert with circulating melatonin to maintain follicular health. He et al. revealed that MT could prevent the apoptosis of porcine GCs during follicular atresia. On the other hand, the atretic follicles exhibit decreased level of MT and increased GCs apoptosis [24]. During maternal aging, the loss of MT in follicular fluid is associated with oxidative stress, compromised oocyte quality, and impaired developmental capacity of ovarian follicles [40,41,42]. These studies suggest a close relationship among stress, MT, and survival of follicular cells. However, it remains unclear whether MT-mediated protection of GCs is disturbed under stress conditions.

Many mammalian species from temperate zones display a seasonal pattern of reproduction controlled by the annual photoperiodic cycle [43]. Depending on the time of year when they are sexually active, species are characterized as short-day breeders, for instance, sheep, or long-day breeders, for instance, hamsters or horses [43]. MT, a pineal hormone that conveys photoperiodic information to the body physiology, has been shown to influence seasonal rhythms in reproductive function [44,45]. In winter, an extended duration of MT synthesis corresponding to prolonged nights stimulates the reproductive axis of short-day breeders and inhibit long-day breeders, illustrating the role that MT plays in a seasonal reproductive state [46,47].

Numerous evidences indicate that MT might improve cellular adaption to hypoxic conditions. Luo et al. reported that MT conferred cardioprotection by inhibiting apoptosis through the activation of the PI3K/Akt signaling pathway in hypoxic cardiomyocytes [48]. In the cytoskeleton, MT has been described to inhibit ROS production induced by hypoxia/reoxygenation (H/R) [49]. Ovarian follicles are a niche enriched with MT derived from the circulation system and follicular cells [38,50]. As such, MT augments the maturation efficiency of oocytes, and it protects the integrity of mouse GCs in pre-ovulatory follicles by reducing oxidative stress [51,52]. Therefore, it prompted us to inquire whether MT contributes to GCs survival in response to hypoxic conditions. Here, we provide the first evidence demonstrating the role of MT in inhibiting hypoxia-induced GCs apoptosis, suggesting that the level of MT in follicular fluid might determine the developmental fate of ovarian follicles.

Moreover, we found that MT protected GCs survival in a dose-dependent manner. MT in concentration at 10^−7^, 10^−5^ M improved GCs viability significantly, while 10^−3^ M suppressed the viability of GC (Figure 1B), indicating that MT at high concentrations has a detrimental effect on GCs under normoxia. However, when GCs were cultured under hypoxia, cell viability was significantly increased by MT at concentrations that ranged from 10^−9^ to 10^−3^ M (Figure 1B). Why did a high concentration (10^−3^ M) of MT exhibit different effects in response to normoxia or hypoxia conditions? One possibility is that hypoxia induced the production of ROS, which reacted with MT; then, it consumed part of MT, hence reducing the cytotoxicity caused by excessive MT.

There are many methods for the detection of cell death. In this study, CCK-8 assay was employed to determine cell viability, Annexin V/PI (propidium iodide) staining was used to measure the cell apoptosis rate, and the cleavage activity of caspase-3 was quantified to assess the intensity of apoptosis. However, our data showed a discrepancy of death rate assessed by viability vs. AnnexinV/PI measurement and AnnexinV/PI staining vs. caspase-3 cleavage. Actually, CCK-8 assay is based on the colorimetric detection of dehydrogenases activity, which is directly proportional to the number of living cells. In another word, CCK-8 could reflect the death rate of cells. Indeed, several forms of cell death have been reported, including autophagic death, apoptosis, necrosis, anoikis, pyroptosis, etc. In addition to apoptosis, it is possible that other forms of cells death occur in GCs suffering hypoxia exposure. Hence, the results of CCK-8 cannot be simply equated with the rate of apoptosis. Although the level of cleaved caspase-3 is a well-described biomarker of apoptosis, the immunoblot technique used for quantitative assessment has limitations [53,54]. Therefore, the quantitative data of cleaved caspase-3 might not be proportional to apoptosis rates. Nevertheless, our results showed similar trend of alteration in apoptosis rate and the caspase-3 cleavage level in GCs with different set of treatments.

Hypoxia has been reported to induce the formation of ROS [55]. Studies revealed that mitochondrion, an organelle required for oxidative phosphorylation, produces a higher level of superoxide through the electron transport chain during hypoxia. In addition, NAD(P)H oxidases might be another generator of ROS under hypoxia conditions [56]. Actually, hypoxia is known to increase oxidative stress, which in turn activates the apoptotic mechanisms [57]. For instance, following cerebral hypoxia, the oxidative damage of mitochondria leads to the release of apoptotic proteins, such as cytochrome c and apoptosis inducible factor (AIF) into the cytosol [34], where cytochrome c binds with AIF and procaspase-9 to form an “apoptosome”, which actives caspase-9 and subsequently caspase-3 cleavage for the execution of the apoptotic program [58]. MT is a well-described antioxidant that exerts its functions by directly scavenging ROS production or indirectly activating the endogenous antioxidant system. As expected, the present study showed that MT treatment could increase the expression of antioxidant enzymes in GCs, decrease the level of ROS, and inhibit hypoxia-induced apoptosis. Our results suggested that the inhibitory effect of MT on GCs apoptosis might partly rely on its antioxidant activity. 

Apart from the antioxidative effects, MT has a bundle of means whereby it thwarts intracellular damage through activating effectors downstream of MT receptors [59]. Previous studies have found that melatonin receptors mainly consist of membrane receptors (MTNR1A, MTNR1B) and nuclear receptors (RZR/ROR, retinoid Z receptor/retinoid acid receptor related orphan receptor) [60]. In ovarian follicles, it has been demonstrated that MT mediated the proliferation, apoptosis, and steroidogenesis of GCs predominantly through the activation of MTNR1B [61,62]. In case of hypoxia, MTNR1B played a protective role in preventing primary cardiomyocytes against hypoxia/reoxygenation injury caused by myocardial ischemia/reperfusion [63]. Here, we described for the first time that in ovarian GCs, MT acts through MTNR1B to inhibit hypoxia-induced apoptosis via silencing the caspase 8/9-caspase 3 axis.

Canonical apoptosis pathways mainly include the mitochondrial apoptosis pathway, death receptor apoptosis pathway, and endoplasmic reticulum apoptosis pathway. Our previous data showed that hypoxia acted through the mitochondrial pathway to induce apoptosis in GCs [30,31]. Consistently, the present study revealed that the mitochondrial pathway, as evidenced by activation of the caspase-9–caspase-3 axis, is required for hypoxia-induced apoptosis in GCs. In agreement with our data, Wang et al. found that melatonin may inhibit GCs apoptosis by degrading Bim_EL_, which is a key component of the mitochondrial apoptosis pathway [64]. In addition, we found that the caspase-8–caspase-3 cascade is concomitantly activated during hypoxia incubation, suggesting that the death receptor apoptosis pathway might also be involved in triggering GCs apoptosis. In addition, the current results showed that MT inhibited the activation of both caspase-8 and caspase-9 upon hypoxia stimulation. Moreover, when GCs were treated with MT, antagonists against caspase-8 or caspase-9 could not further inhibit caspase-3 cleavage, indicating that MT can inhibit caspase-3 activation by inhibiting the mitochondrial apoptosis pathway and death receptor apoptosis pathway.

## 5. Conclusions

**Figure 6:**
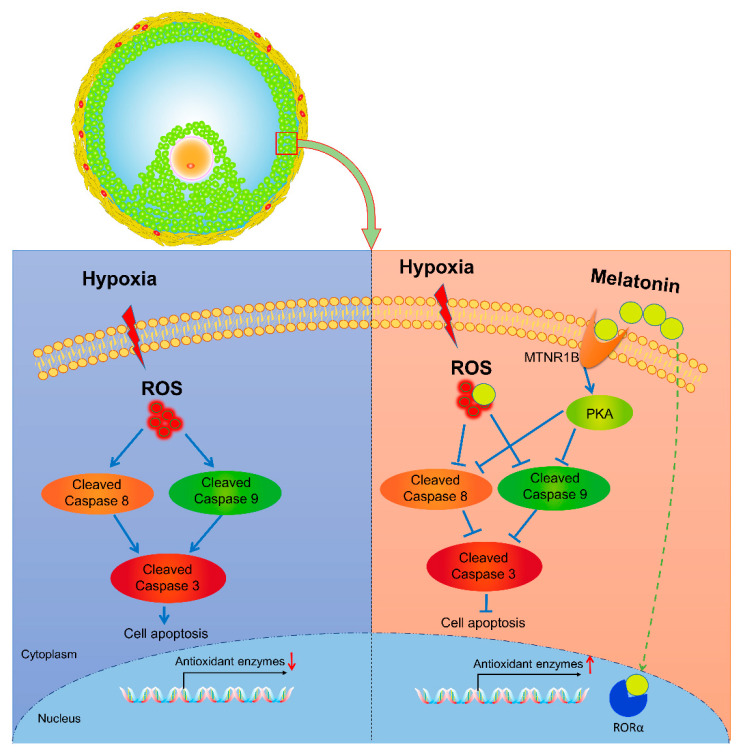
Schematic illustration of the signaling pathway involved in MT-mediated GCs resistance to hypoxia-induced apoptosis. Hypoxia facilitates ROS accumulation and the cleavage of the initiator caspases (8, 9), which in turn activates the executioner caspase 3, thereby inducing GCs apoptosis. In the presence of MT, the apoptosis of GCs is inhibited, possibly through the clearance of cellular ROS, activation of antioxidant enzymes expression, or suppression of Caspase 8/9-Caspase 3 axis via the MT–MTNR1B–PKA pathway.

In conclusion, the current research uncovered a novel role of MT in repressing hypoxia-induced GCs apoptosis through scavenging cellular ROS, stimulating antioxidant enzymes expression, or inhibiting the caspase 8/9–caspase 3 axis via the MTNR1B–PKA pathway. These findings not only extended our understanding regarding the mechanism of follicular development under hypoxia but also provided potential avenues for improving animal reproductive performance by supplementing MT (Figure 6).

## Figures and Tables

**Table 1:** Primers for qRT-PCR.

Genes	Primer Sequence (5′–3′)	Product Size (bp)	Accession No.
*GAPDH*	Forward: GTCGGTTGTGGATCTGACCT	207	XM_021091114.1
	Reverse: TTGACGAAGTGGTCGTTGAG		
*ACTB*	Forward: TGCGGGACATCAAGGAGAAGC	273	XM_021086047.1
	Reverse: ACAGCACCGTGTTGGCGTAGAG		
*HO-1*	Forward: CCGAGAAGGCTTTAAGCTGGT	110	NM_001004027.1
	Reverse: GGAAGTAGAGGGGCGTGTAG		
*TRX1*	Forward: CTTTACCTTATTGCCCGGGT	162	NM_214313.2
	Reverse: GTTCACCGATTTTGTTGGCC		
*GST*	Forward: CAACCCAGAAGACTGCTCAAG	159	NM_214300.1
	Reverse: GGACCACTCAAGGAATACAGAAG		
*SOD1*	Forward: TCCATGTCCATCAGTTTGGA	248	NM_001190422.1
	Reverse: AGTCACATTGGCCCAGGTCTC		
*SOD2*	Forward: AGGCGCTGAAAAAGGGTGAT	163	NM_214127.2
	Reverse: AAGTCGCGTTTGATGGCTTC		
*CAT*	Forward: CACACATACCCATTCGTCACT	157	NM_214301.2
	Reverse: CAGCCCTAACCTTCACTTACC		
*GPX4*	Forward: ATTCTCAGCCAAGGACATCG	93	NM_214407.1
	Reverse: CCTCATTGAGAGGCCACATT		
*FAS*	Forward: CCTGCCATCCCTGATGCTATT	156	XM_021072073.1
	Reverse: AGCGATACAGGTAAATACATGGC		
*FASL*	Forward: GAGAGTCTGCCAGCCAAAGG	125	NM_213806.1
	Reverse: TCTTGAGTTAGGCTTGCCTGTT		
*TNFR1*	Forward: ACCGGGAGAAGAGAGAGAGTT	102	NM_213969.1
	Reverse: TGTGTAGGTAGGTGCCTTTGTG		
*TRAIL*	Forward: GGGCAGACCTGTGTGTTGAT	114	NM_001024696.1
	Reverse: GGAGTACTTGTCCTGCATCTGT		

## Data Availability

Data available in a publicly accessible repository.
